# Obesity and unhealthy lifestyle associated with poor executive function among Malaysian adolescents

**DOI:** 10.1371/journal.pone.0195934

**Published:** 2018-04-17

**Authors:** Joyce Ying Hui Tee, Wan Ying Gan, Kit-Aun Tan, Yit Siew Chin

**Affiliations:** 1 Department of Nutrition and Dietetics, Faculty of Medicine and Health Sciences, Universiti Putra Malaysia, Serdang, Selangor, Malaysia; 2 Department of Psychiatry, Faculty of Medicine and Health Sciences, Universiti Putra Malaysia, Serdang, Selangor, Malaysia; 3 Research Centre of Excellence for Nutrition and Non-Communicable Diseases, Faculty of Medicine and Health Sciences, Universiti Putra Malaysia, Serdang, Selangor, Malaysia; Pondicherry Institute of Medical Sciences, INDIA

## Abstract

The understanding on the roles of obesity and lifestyle behaviors in predicting executive function of adolescents has been limited. Low executive function proficiency may have adverse effects on adolescents’ school academic performance. This cross-sectional study aimed to examine the relationship between BMI-for-age and multiple lifestyle behaviors (operationalized as meal consumption, physical activity, and sleep quality) with executive function (operationalized as inhibition, working memory, and cognitive flexibility) on a sample of Malaysian adolescents aged between 12 and 16 years (*N* = 513). Participants were recruited from two randomly selected schools in the state of Selangor in Malaysia. Using a self-administered questionnaire, parent participants provided information concerning their sociodemographic data, whereas adolescent participants provided information regarding their meal consumptions, physical activity, and sleep quality. The modified Harvard step test was used to assess adolescents’ aerobic fitness, while Stroop color-word, digit span, and trail-making tests were used to assess adolescents’ inhibition, working memory, and cognitive flexibility, respectively. Three separate hierarchical regression analyses were conducted for each outcome namely, inhibition, working memory, and cognitive flexibility. After adjusted for sociodemographic factors and BMI-for-age, differential predictors of inhibition and working memory were found. Habitual sleep efficiency significantly and positively predicted inhibition. Regular dinner intakes, physical activity levels, and sleep quality significantly and positively predicted working memory. Household income emerged as a consistent predictor for all executive function domains. In conclusion, an increased trend of obesity and unhealthy lifestyles among adolescents were found to be associated with poorer executive function. Regular dinner intakes, higher physical activity levels and better sleep quality predicted better executive function despite the inverse relationship between obesity and executive function. Future studies may explore how lifestyle modifications can optimize the development of executive function in adolescents as well as relieve the burden of obesity.

## Introduction

During the transitional stage between childhood to adulthood (henceforth referred to as adolescence), individuals undergo considerable physiological, psychosocial, and cognitive development changes [1]. The changes are further accompanied by elevations in sensation and exploration of new things. The elevations mentioned may promote an enthusiasm for learning and adapting. Conversely, the elevations may result in increased vulnerability to risk-taking behaviors [2]. In the transitional stage from childhood to adulthood, individuals’ success in accomplishing developmental tasks highly depends on their self-regulatory abilities, namely executive function (EF).

The EF is governed by the prefrontal cortex (PFC). The EF is a cluster of cognitive processes that underlie planning, organizing, and regulation. Thus, it is responsible for the achievement of purposeful, goal-directed behaviors [3]. According to the concept of “unity and diversity of EF” [4], EF consists of three interrelated but distinct domains. The first, known as inhibition, concerns the ability to resist distraction and to maintain focus. The second, working memory (WM), is related to the ability to store, maintain, and manipulate information over a brief period of time. The third, cognitive flexibility (CF), concerns the ability to shift attention, select information, and alter response strategies in response to changing task demands. Findings from previous studies have suggested that inhibition, WM, and CF share a few common underlying processes. Nonetheless, the three domains are dissociable when measured using different neurocognitive tests [5]. The domains of EF are crucial for the lifelong wellbeing of adolescents. Not only does EF serve as ground support for contemporaneous learning capabilities such as time management, it also promotes future learning capabilities and career success [6]. The role of EF has also been attributed to negative health outcomes, in which EF deficits were associated with psychosocial problems such as aggression, externalizing and internalizing behaviors [7]. Undeniably, EF affected physical, mental, social, and psychological well-being of individuals from childhood to adolescence and then subsequently adulthood [8].

There has been increased research on the relationship between obesity and EF, which in line with the rising global burden of childhood obesity [9]. Growing body of evidence linked obesity to adverse neurodevelopmental outcomes in adults, such as impaired grey and white matter of the brain and the degeneration of PFC regions which has led to poor EF [10]. It was found that even where there was an absence of degenerative effects of the aging process and other obesity co-morbidities, subtle brain alterations with lower EF levels were uncovered among obese adolescents [11]. Previous studies have shown a consistent relationship between a high body mass index (BMI) and poor inhibition among adolescents [10]. Nevertheless, the relationship between obesity and other domains of EF have remained under-examined and findings for such a relationship have been inconclusive [12].

A ‘healthy lifestyle behavior’ is a broad term encompassing behaviors such as being physically active, not smoking, moderate consumption of alcohol, and healthy dietary intakes. Healthy lifestyle behaviors have been shown to facilitate protection against dementia and cognitive decline among older adults [13]. The cognitive benefits of adopting a healthy lifestyle may not be restricted to adults. In fact, some lifestyle behaviors such as skipping breakfast, physical inactivity, and sleep deprivation were commonly observed in adolescents. Moreover, such behaviors persisted when the adolescents became adults [14]. Extensive studies have reported that unhealthy lifestyle behaviors could potentially deteriorate academic [15] and cognitive performances, such as academic-related skills [16], and intelligence quotients (IQ) [17]. Nevertheless, a few studies have simultaneously examined the relationship between multiple lifestyle behavior domains and EF in adolescents.

It has been acknowledged that the lifestyle behavior, for instance *breakfast consumption* has benefited the memory and attention of learning for children and adolescents. Nonetheless, studies on EF have yielded mixed results [18, 19]. Moreover, previous studies have yet to take into account the intakes of lunch and dinner, which could be important contributors to daily nutritional intakes. A review paper suggested that *physical exercise* positively improved EF, particularly notable for inhibition domain [20]. It was also found that aerobic fitness enhanced inhibition [21]. On the other hand, there was a study that showed a lack of correlation between daily physical activities and EF domains [22]. If the studies are viewed collectively, previous scholars were often focused on a single domain of EF and their findings were inconsistent. Another lifestyle behavior, *sleep deprivation* has been found to correlate with cognitive functions [22] and affected school performance [16] of adolescents. The heavy burden of academic work has resulted in a drastic drop in the sleep duration of children and adolescents since the 20^th^ century. Indeed, the highest rate of sleep reduction was within the Asian population [23]. Despite this, there is a lack of data available on the relationship between sleep deprivation and EF in adolescents in Asian countries.

From a general perspective, obesity and unhealthy lifestyle behaviors could plausibly contribute to poor EF in adolescents. The transitional stage from childhood to adulthood could be a critical period to ensure optimal EF development and thus conferring cognitive stability thereafter. Nonetheless, the primary focus of EF research to date has been focused on only obesity or single lifestyle behaviors. The relationship between obesity and multiple lifestyle behaviors with EF remains under-explored. Therefore, the objective of the present study was to examine the relationships between obesity and several lifestyle behaviors of adolescents (operationalized as meal consumption patterns, physical activity, fitness levels, and sleep quality) with EF, taking account the influences of socio-demographic factors.

## Materials and methods

### Study design and population

The present research was a cross-sectional study which targeted adolescents aged between 12 and 16 years old. A probability proportionate to size method was utilized and two secondary schools in the state of Selangor, Malaysia were randomly selected. Adolescents who suffered from neurological or psychiatric disorders (e.g., autism spectrum disorders, anxiety, and depression), had medical conditions (e.g., sleep disorders, diabetes, thyroid disease, and cardiovascular diseases), had learning disabilities or developmental delays, had a history or presence of traumatic brain injuries, and had difficulties in performing physical activities and anthropometric measurements due to pre-existing illnesses were excluded from this study. There were 560 eligible adolescents and 538 agreed to participate and completed the study questionnaire. A total of 513 adolescents were retained in the final analysis due to a number of parent participant questionnaires not being returned and a few adolescents were moved to other schools. Consequently, the response rate was 92%.

Prior to the collection of data, ethical approval was obtained from the Ethics Committee for Research Involving Human Subject of Universiti Putra Malaysia [Reference No. FPSK(EXP16) P186]. Permission to conduct the study was also obtained from the Ministry of Education, Selangor Department of Education and from the authorities of the selected schools. Several visits to the respective schools were carried out. On the first visit, all eligible participants were briefed on the study’s objectives and the activities they would participate in. The eligible participants were provided with an information sheet. Parent and adolescent consent forms as well as a set of questionnaire for parent participants requesting the provision of sociodemographic information were issued to be taken home by adolescent participants. The completed forms were collected the next day. In subsequent visits, adolescent participants who returned the parent and adolescent consent forms were guided to answer a self-administered questionnaire regarding lifestyle behaviors on meal consumption patterns, physical activity, and sleep quality. The adolescent participants also had their anthropometric measurements taken and underwent an aerobic fitness test. The researchers received training from a clinical psychologist from the Universiti Putra Malaysia to assess the adolescent participants’ EF using three neurocognitive tests in an empty classroom provided by the school authorities.

### Neurocognitive tests

The construct of EF was shown by three main domains, namely inhibition, WM and CF [6]. The domains were measured by a specific neurocognitive test. Inhibition was assessed using the Stroop color-word test [24] which consisted of three trials: (i) word tasks (participants read color names printed in black ink), (ii) color tasks (participants read colors of Xs printed in green, red, or blue), and (iii) word-color tasks (participants named the color of the ink which the words were printed, ignoring the words that were printed). There were 5 columns of 20 items, totaling 100 items per trial. The adolescent participants in this study were instructed to read items from the top to the bottom of the columns for 45 seconds. The number of items correctly named were calculated as a raw score for each trial. Based on the formula provided in the test manual [24], the interference score for each participant was calculated from the raw scores, where the scores ranged between -50 to 37. Higher interference scores indicated a better capacity of inhibition responses.

Working memory (WM) was assessed using the Digit Span test from the Wechsler Intelligence Scale for Children-Fourth Edition [25]. The test was divided into two parts: Digit Span Forward and Digit Span Backward. Each part contained 8 items with 2 trials per item. In each trial, participants recited a string of digits at the rate of one digit per second. The participants were required to repeat the string of digits as they heard it (Digit Span Forward) or in a reverse manner (Digit Span Backward). Each trial had a digit length that began with 2 digits which increased sequentially to 9 digits until recall errors were made for at least both trials of an item. The participants in this study were awarded one point for every correct repeated digit string and no points for recall errors. The points for both parts were summed up to generate a total score. Higher scores indicated a better WM performance.

Cognitive flexibility (CF) was measured using a Trail-making test (TMT) [26]. The TMT consisted of Trail-making A and B. In Part A, participants were given a piece of paper containing 25 randomly arranged, numbered circles. The participants were then instructed to draw lines between the circles in an ascending order. In Part B, participants were given another piece of paper containing 13 numbers (1–13) and 12 alphabets (A-L), which were intermixed. The participants were asked to draw lines connecting the numbers and alphabets alternately in a sequential order (i.e., 1-A-2-B-3-C etc.). They were then instructed to connect the numbers and alphabets as fast as possible. The time to complete both parts were recorded, including the time it took to make corrections if participants made errors when connecting the trails. A task-switching score was calculated by subtracting the time it took to complete Part A from Part B. A shorter time indicated a better CF. In other words, a lower score denoted a better performance.

### Self-administered questionnaires

Parent participants completed a Malay-English bilingual questionnaire which contained a provision of sociodemographic characteristics such as monthly household incomes, household sizes, highest education levels, and number of years of education attained. On the other hand, using another self-administered questionnaire, adolescents were requested to provide information regarding their age, date of birth, sex, ethnicity, meal consumption patterns, physical activity, and sleep quality.

#### Meal consumption patterns

An Eating Behaviors Questionnaire (EBQ) was used to measure frequency of main meal intakes and snacking behavior of the adolescent participants [27]. The participants reported the number of days per week (0–7 days) they had their main meals and snacks. The operational definitions of meal intakes were specified in the questionnaire as follows: ***Breakfast*** referred to the first meal of the day which covered any food or beverages (except plain water) consumed before 10 AM. ***Lunch and dinner*** referred to meals taken between 11 AM to 3 PM and after 5 PM [27]. ***Snacking*** was defined as the consumption of any food or beverages (except plain water) between the three main meals [28]. Adolescent participants were considered to skip breakfast/lunch/dinner if they consumed main meals fewer than 7 days per week. Moreover, those who had main meals every day were considered to have a regular breakfast/lunch/dinner intake [28]. The internal consistency of EBQ in this study was acceptable (Cronbach’s alpha = 0.60).

#### Physical activity levels

The Physical Activity Questionnaire for Older Children (PAQ-C) was used to measure adolescents’ physical activity levels for one week [29]. The questionnaire had ten items, the first nine items covered activities during physical education classes, leisure time, recess, lunch times, evenings, and weekends, as well as the frequency of daily physical activities. The last item covered unusual activities that prevented adolescents from carrying out their regular activities. Each item was scored using a 5-point scale. Higher scores indicated a higher level of physical activity. The summary activity score was calculated from the mean score of the nine items to classify adolescents into different levels of physical activity: low (1.00–2.33), moderate (2.34–3.66) and high (3.67–5.00) [30]. The internal consistency of PAQ-C in this study was excellent (Cronbach’s alpha = 0.902).

#### Sleep quality

Adolescents’ sleep quality was measured over one month using the Pittsburgh Sleep Quality Index (PSQI) [31]. The index had 19 items encompassing 7 components which were: subjective sleep quality, sleep latency, sleep duration, habitual sleep efficiency, sleep disturbance, use of sleep medication, and daytime dysfunction (sleepiness). The frequency of sleep problems were rated using a scale from 0 (*not during the past month*) to 3 (*2–3 times per week*). The length of time it took for participants to wake up and sleep on weekdays and weekends were reported for their sleep duration calculation. Adolescents could be classified as short sleepers (i.e., < 9 hours) or normal sleepers (i.e., ≥ 9 hours) [32]. A habitual sleep efficiency percentage was generated (actual sleep duration/number of hours spent in bed × 100%) to determine the percentage of actual sleep gained. The percentage excluded the time spent in bed where a participant remained sleepless. A global sleep quality score (ranged 0 to 21) was generated from the sum of 7 component scores. Higher scores indicated a bad sleep quality. If a participant’s global sleep quality score was less than 5, it indicated they had good sleep quality. On the other hand, a score ≥ 5 indicated poor sleep quality. The internal consistency of PSQI in the current study was good (Cronbach’s alpha = 0.757).

### Anthropometric measurements

All measurements were collected twice to obtain the mean values for data analysis. Body weight (0.1 kg precision) and height (0.1 cm precision) of the adolescent participants were measured in light clothing, with no shoes. A TANITA digital weighing scale THD-306 (TANITA Corporation, Arlington Heights, IL, USA) and a SECA portable stadiometer 213 (SECA, Hamburg, Germany) were used to carry out the measurements. The Body Mass Index for age (BMI-for-age) z-score was calculated using the World Health Organization (WHO) AnthroPlus software version 1.0.4 (WHO, Geneva, Switzerland) to classify respondents into several categories of body weight status according to the WHO Growth Reference 2007 [33].

### Aerobic fitness

The step test was recommended as the more practical option for mass testing of aerobic fitness compared to the direct measure of VO_2_ max which involved sophisticated laboratory procedures [34]. All the participants performed the modified Harvard Step Test in their sports attire following a standardized protocol [35]. Physical fitness scores (PFS) were derived by taking the total duration of exercise in seconds × 100 and dividing it with the sum of three heart rates at 0, 1 and 2 minutes after the cessation of the exercise protocol [36]. The PFS was used to classify the aerobic fitness levels of the participants. A PFS score < 55 was considered to be poor, whereas a score of 55–64 was considered to be a low average. A score of 65–79 was deemed to be a high average, 80–89 was good, and > 90 was excellent [35].

### Statistical analysis

All analyses were performed using the IBM SPSS Statistics 24 (IBM Corp., Armonk, NY, USA). The relationships between socio-demographic factors, BMI-for-age, lifestyle behaviors, and EF were inspected using a Pearson product-moment correlation test. Three separate hierarchical multiple regression analyses were conducted on each EF domain (inhibition, WM, and CF). A proximal-distal approach was applied to determine the order to enter the study variables into the model. Socio-demographic factors were treated as distal factors as they did not exert direct influences on EF. Modifiable factors were treated as proximal factors. In order to determine the differential contribution of each block of variables towards the prediction of EF, socio-demographics (age, sex, monthly household income, parents’ years of education, and household size) were entered in the first block. The BMI-for-age followed in the second block and then all lifestyle behaviors (meal intakes, sleep quality, physical activity and aerobic fitness) were placed in the last block. The level of significance for all tests was set at *p* < 0.05.

## Results

Characteristics of the participants are outlined in Table 1. Out of 513 adolescents, 41.1% were males and 58.9% were females. The mean age for the participants was 14.08 (*SD* = 1.32) years. A majority of the participants came from families with monthly household incomes below RM5600 (72.4%). Most of the participants fathers (60.3%) and mothers (59.7%) had attained a secondary education. One third of the adolescents (32.6%) in this study were overweight or obese. Unhealthy lifestyle behaviors were also prevalent in the adolescents. Around 70.0% of the adolescents reported that they skipped breakfast, while 47.2% skipped lunch and 47.4% skipped dinner. Only 6.2% the adolescents were found to engage in high levels of physical activity. Similarly, only 9% of the adolescents had acceptable levels of aerobic fitness (PFS ≥ 65). More than half of the adolescents did not have 9 hours of daily sleep (68.0%), while 72.5% were classified as having poor sleep quality.

**Table 1:** Characteristics of the participants (*n* = 513).

Variables	n (%)	Mean ± SD
**Age (years)**		14.08 ± 1.32
**Sex**		
Male	211 (41.1)	
Female	302 (58.9)	
**Ethnicity**		
Malay	303 (59.1)	
Chinese	49 (9.6)	
Indian	135 (26.3)	
Others	26 (5.0)	
**Household sizes (members)**		
≤ 5	267 (55.0)	
≥ 6	219 (45.0)	
**Monthly household income (RM)**		
< RM2300.00	188 (38.7)	
RM2300.00 –RM5599.00	164 (33.7)	
≥ RM5600.00	134 (27.6)	
**Mother number of years in education**		13.01 ± 3.72
Secondary education and below	319 (65.6)	
Tertiary education	167 (34.4)	
**Father number of years in education**		13.09 ± 3.66
Secondary education and below	321 (66.0)	
Tertiary education	165 (34.0)	
**BMI-for-age z-score**		0.25 ± 1.52
Thinness	31 (0.6)	
Normal	311 (60.6)	
Overweight	93 (18.1)	
Obesity	74 (14.5)	
**Meal consumption patterns (intake per week)**		
**Breakfast intakes**		4.18 ± 2.39
Daily	159 (31.0)	
< 7 days per week	354 (69.0)	
**Lunch intakes**		5.43 ± 2.01
Daily	271 (52.8)	
< 7 days per week	242 (47.2)	
**Dinner intakes**		5.27 ± 2.25
Daily	270 (52.6)	
< 7 days per week	243 (47.4)	
**Physical activity levels**		2.56 ± 0.64
Low (1.00–2.33)	199 (38.8)	
Moderate (2.34–3.66)	282 (55.0)	
High (3.67–5.00)	32 (6.2)	
**Aerobic fitness levels**		62.96 ± 12.01
Poor (<55)	110 (21.4)	
Low average (55–64)	218 (42.5)	
High average (65–79)	139 (27.1)	
Good (80–89)	29 (5.7)	
Excellent (>90)	17 (3.3)	
**Sleep quality**		6.23 ± 2.51
Poor quality (> 5)	372 (72.5)	
Good quality (≤ 5)	141 (27.5)	

Note. RM = Ringgit Malaysia

### Correlations of BMI-for-age, lifestyle behaviors, and EF

Table 2 demonstrates that BMI-for age was negatively correlated with adolescents’ performance of inhibition (*r* = -0.096, *p* = 0.029) and WM (*r* = -0.098, *p* = 0.027). Nonetheless, there was not a significant correlation detected for CF (*r* = 0.038, *p* = 0.387). Regular meal intakes were positively correlated with WM and CF. Physical activity trended towards a positive correlation with WM (*r* = 0.086, *p* = 0.052), but it was not significant. Aerobic fitness did not correlate with any EF domains. Weekdays sleep duration correlated negatively with WM (*r* = -0.102, *p* = 0.021) and CF (*r* = -0.116, *p* = 0.009). It was found that adolescents who slept less than 9 hours tended to score better compared to those who had at least 9 hours of sleep during weekdays. Nevertheless, a significant correlation was not found for weekend sleep durations. Instead, habitual sleep efficiency was found to be positively correlated with inhibition (*r* = 0.123, *p* = 0.005) and WM (*r* = 0.097, *p* = 0.028). Furthermore, better sleep quality (lower score) was significantly correlated with higher WM (*r* = -0.218, *p* < 0.001).

**Table 2:** Pearson-product moment correlations between BMI-for-age and lifestyle behaviors with EF.

Variables	Interference score		WM total score		Task-switching score	
	*r*	*p*	*r*	*P*	*r*	*p*
BMI-for-age z-scores	-0.096*	0.029	-0.098*	0.027	0.038	0.387
Breakfast intakes	0.025	0.577	0.134**	0.002	-0.011	0.795
Lunch intakes	-0.007	0.877	0.166**	<0.001	-0.109*	0.013
Dinner intakes	0.075	0.092	0.190**	<0.001	-0.099*	0.025
Physical fitness scores	0.032	0.464	-0.010	0.813	0.063	0.151
Physical activity levels	-0.069	0.120	0.086	0.052	0.021	0.638
Habitual sleep efficacy	0.123*	0.005	0.097*	0.028	-0.039	0.383
Sleep quality scores	-0.024	0.588	-0.218**	<0.001	0.045	0.306
Weekday sleep durations	-0.033	0.462	-0.102*	0.021	0.116**	0.009
Weekend sleep durations	-0.073	0.098	0.024	0.590	0.005	0.904

Note. EF = Executive Function; WM = Working Memory

*correlation is significant at *p* < 0.05

**correlation is significant at *p* < 0.01

### Predictors of EF domains

Tables 3–5 summarized the results of the hierarchical regression models for each EF domain. The addition of multiple lifestyle behaviors increased the overall fit of all three models. Moreover, several variables were found to explain significant, unique variances in the EF domains. Monthly household income consistently appeared as a significant predictor of all three domains. Adolescents from higher income families (≥RM5600) showed higher EF compared to those who came from lower income families (<RM5600). BMI-for-age (*β* = -0.097, *p* = 0.033) remained as a significant predictor of inhibition after socio-demographic variables were controlled. It was also found that habitual sleep efficiency (*β* = 0.106, *p* = 0.019) positively predicted inhibition. Fathers’ years of education (*β* = 0.121, *p* = 0.017) and household size significantly predicted WM (*β* = -0.101, *p* = 0.024). After controlling socio-demographic variables, BMI-for-age (*β* = -0.114, *p* = 0.010) negatively predicted WM (Δ*R*^*2*^ = 0.013). It was also found that sleep quality (*β* = -0.196, *p* < 0.001), dinner intakes (*β* = 0.091, *p* = 0.037) and physical activity levels (*β* = 0.097, *p* = 0.028) positively predicted WM of adolescents (Δ*R*^*2*^ = 0.064). In addition to income, CF was significantly predicted by age, sex and household size (Δ*R*^*2*^ = 0.046). As expected, older ages of adolescents were associated with a higher CF (*β* = -0.144, *p* = 0.002). In comparison to females, males were found to have significantly better CF (*β* = 0.110, *p* = 0.015). Greater household sizes were associated with lower CF (*β* = 0.089, *p* = 0.047). Nevertheless, none of the lifestyle behaviors significantly predicted CF in the final model after socio-demographic and BMI-for-age factors were added.

**Table 3:** Summary of hierarchical regression analyses predicting adolescents’ inhibition.

Predictors	*β*	*ΔR*^*2*^
**Step 1**		0.012
Age	0.037	
Father number of years in education	-0.070	
Monthly household income	0.121*	
**Step 2**		0.009
Age	0.038	
Father number of years in education	-0.066	
Monthly household income	0.129*	
BMI-for-age	-0.097*	
**Step 3**		0.011
Age	0.038	
Father number of years in education	-0.065	
Monthly household income	0.127*	
BMI-for-age	-0.095*	
Habitual sleep efficiency	0.106*	
***R***^***2***^***(Full model)***		0.033

Inhibitory control: *F* (5,485) = 3.274, *p*<0.01

*p<0.05,

**p<0.01,

***p<0.001

Dummy coding: Monthly household income (<RM5600 = 0, ≥RM5600 = 1)

**Table 4:** Summary of hierarchical regression analyses predicting adolescents’ working memory.

Predictors	*β*	*ΔR*^*2*^
**Step 1**		0.051
Age	0.088	
Father number of years in education	0.121*	
Household size	-0.101*	
Monthly household income	0.110*	
**Step 2**		0.013
Age	0.089*	
Father number of years in education	0.126*	
Household size	-0.100*	
Monthly household income	0.119*	
BMI-for-age	-0.114*	
**Step 3**		0.064
Age	0.127**	
Father number of years in education	0.116*	
Household size	-0.071	
Monthly household income	0.120*	
BMI-for-age	-0.083	
Frequency of dinner intakes	0.091*	
Global sleep quality	-0.196***	
Physical activity	0.097*	
***R***^***2***^***(Full model)***		0.128

Working memory: *F* (9,485) = 7.753, *p*<0.001

*p<0.05,

**p<0.01,

***p<0.001

Dummy coding: Monthly household income (<RM5600 = 0, ≥RM5600 = 1), Physical activity (low & moderate = 0, high = 1)

**Table 5:** Summary of hierarchical regression analyses predicting adolescents’ cognitive flexibility.

Predictors	*β*	*ΔR*^*2*^
**Step 1**		0.046
Age	-0.144**	
Sex	0.110*	
Household size	-0.089*	
Monthly household income	0.088*	
**Step 2**		0.002
Age	-0.145**	
Sex	0.108*	
Household size	0.088*	
Monthly household income	-0.092*	
BMI-for-age	0.039	
**Step 3**		0.014
Age	-0.123	
Sex	0.074	
Household size	0.069	
Monthly household income	-0.068	
BMI-for-age	0.048	
Frequency of lunch intake	-0.053	
Frequency of dinner intake	-0.063	
Weekdays sleep duration	0.063	
Physical fitness score	0.060	
***R***^***2***^***(Full model)***		0.061

Cognitive flexibility: *F* (9,485) = 3.452, *p*<0.001

*p<0.05,

**p<0.01

Dummy coding: Sex (male = 0, female = 1), Monthly household income (<RM5600 = 0, ≥RM5600 = 1), Weekdays sleep duration (<9 hours = 0, ≥9 hours = 1)

## Discussion

Evidence linking overweight and obesity to EF in adolescents has been accumulating [9, 37– 39]. Nonetheless, the extent that multiple lifestyle behaviors could have effects on EF after controlling the influence of obesity has remained largely unexplored. To the best of the researcher’s knowledge, this is the first study to examine the relative contributions of socio-demographic factors, BMI-for-age, and lifestyle behaviors on EF, in a sample of Malaysian adolescents.

The current study found that monthly household income consistently appeared as a significant predictor for all EF domains. The findings were in line with previous studies which showed that children and adolescents from higher income families performed better in the neurocognitive testing of EF [40,41]. It has been reported in recent neurostructural data that low family income and reduced brain integrity and volumes were predictive of poor EF among children and adolescents [42]. The present study demonstrated that larger household sizes predicted poorer WM and CF. The findings supported Blair et al’s study [43], which similarly found that household density correlated negatively with EF. The present study’s findings were also in line with Mohd Nasir et al’s study [44], which showed that the higher number of siblings negatively predicted cognitive performances in young children. Large household sizes and low family income levels could be a proxy for low socio-economic status (SES). Children from poor SES backgrounds may experience impaired family functions and have limited access to resources that foster cognitive skills [45]. As a result, it is suggested that adverse environments could pose a threat to children’s mental and psychosocial development.

The current study found that a father’s educational level positively predicted WM. Numerous studies have found positive relationships between maternal or parental education with EF among younger children aged below 13 years [40–41,46]. Nonetheless, previous studies have not investigated the differential effects of maternal and paternal education levels on EF. The present study provides preliminary evidence for the role of paternal education in explaining EF development. According to previous literature, women who received a higher education were empowered with knowledge and skills required for employment and child bearing. The skills enabled them to provide a home environment that actively stimulated children’s cognitive and emotional growth [47]. However, the effect of maternal education on EF was only apparent during childhood and seemed to slowly reduce in adolescence as other environmental influences such as schooling, neighborhood environment and social interactions played a bigger role [40,46]. In contrast, recent longitudinal data revealed that paternal education could have a pervasive effect on the achievements of children [48]. Although the underlying mechanisms are still uncertain, it is possible that fathers with a higher education may plausibly promote better acquisition of EF in adolescents through positive parenting behaviors, a higher quality of family interactions, and better living and learning resources [49].

After sociodemographic influences were taken into consideration, the present study identified a significant relationship between high BMI-for-age and poor inhibition and WM in adolescents. The findings were consistent with findings in previous studies [37,38]. It was found that overweight and obese adolescents may have greater difficulties in suppressing irrelevant automated responses, directing attention, and manipulating and holding information in their minds [38]. Similar to the findings in previous studies, [11,50], the association between BMI-for-age and CF was not significant in the present study. Conversely, other studies demonstrated that increased BMI predicted cognitive inflexibility in relatively small samples of adolescents with a large proportion of obese individuals (> 40%) [37,39]. It was plausible that the deteriorating effects of adiposity on CF were more likely to be detectable over a threshold and perhaps only in a large proportion of an obese sample of individuals [51].

Lifestyle behaviors were also predictive of EF development during adolescence. After the BMI-for-age factor was controlled, meal intakes, physical activity, and sleep quality emerged as significant predictors of EF. The present study found that regular meal intakes throughout a day and especially dinner intakes were found to predict better WM and CF. The finding was in line with Mohd Nasir et al.'s [44] study which documented that regular dinner consumption was a significant contributor to general cognitive abilities of 1,933 Malaysian preschoolers. It is important to note that regular meal intake finding was not conclusive. Dinner was found to be the most important meal for Malaysians, contributing to a low meal skipping rate. For adults, dinner contributed to high proportion of calorie intakes per day [52]. Similarly, irregular intakes of lunch and dinner, but not breakfast, contributed significantly to lower intakes of macronutrients and total energy among Malaysian children and adolescents [28]. Such findings may plausibly suggest that adolescents who skipped dinner could be at risk of inadequate macronutrients. Macronutrients such as glucose are main substrates of brain cells and can thus affect the function of PFC that governs EF.

Other than meal skipping, sleep deprivation was another pervasive habit observed in adolescents in this study. The present findings are similar to previous findings [53,54] identified that sleep parameters including habitual sleep efficiency and sleep quality were related to WM and inhibition. Adolescents in this study who had shorter sleep durations (< 9 hours) performed surprisingly better for WM and CF compared to those who had longer sleep durations (≥ 9 hours). A systematic review of sleep studies among children and adolescents suggested that optimal sleep duration for those aged 10 years and above was at least 9 hours sleep per day [32]. It was acknowledged that longer durations of sleep may be related to higher EF [55]. Nevertheless, the recommendation of 9 hours of sleep per day might not be relevant for Asian countries including Malaysia, due to a great diversity in sleep patterns across countries [56]. In fact, it has been found that bedtime behaviors and sleep duration were affected by cultural practices including homework ethics [57]. It could be possible that Asian adolescents required less sleep than Caucasians due to cultural factors, although more investigation is needed. Moreover, a neuroimaging study by Telzer et al. [58] found that poorer sleep quality was correlated with less activation of dorsolateral PFC but higher insula activation in adolescents. Telzer et al’s [58] finding suggested that adolescents who had poor sleep quality were prone to low vigilance in decision making and a high orientation of risk taking.

The present study reported that better WM was predicted by a higher level of PA in adolescents. The finding echoes previous findings [59,60]. Although there was a cognitive benefit of PA on inhibition, its effect was rather short-term, as the cognitive measurements were performed just after a single bout of acute exercise [20]. In fact, chronic exercise was more likely to demonstrate long-term beneficial effects [60]. The benefits were brought about by permanent physiological changes in the brain, such as the formation and extension of new vessels and the release of neurotrophic factors that enhanced neural growth and survival. All together the physiological changes induced brain cognitive reserves. Padilla et al. [60] found that the association between PA and inhibition was no longer significant after WM was controlled as a covariate, suggesting the substantial contribution of WM in inhibitory processes. In fact, inhibition and WM have been consistently found as independent, yet interrelated domains of EF that shared limited brain resources to perform the tasks [4,61]. Thus, the performance of inhibitory tasks was likely to depend on the interaction of both domains for resource sharing and the level of task demands on each domain [61]. This implies that the individual differences in performing a task is observable only when task demands for WM and inhibition exceed a certain threshold. Plausibly, the task that was used to measure inhibition (i.e.; the Stroop test), required less WM resources. Thus, a significant relationship between PA and inhibition could not be observed.

The present study did not find a significant association between aerobic fitness and inhibition, which replicated previous findings that similarly used SCWT as a measure of inhibition [62]. Nonetheless, the results were in contradiction with other studies that applied a flanker task [63, 64]. The inconsistent findings could be due to the variation in tests of EF, aerobic fitness and the adjustment of confounders across studies. It is undeniable that the relationship between aerobic fitness and EF can still be debated. It has been suggested that people who exercise regularly and intensively tend to be more fit and have higher EF [64]. Nevertheless, evidence on this suggestion has not been conclusive, as aerobic fitness was partly determined by genetics and may not adequately reflect one’s level of daily physical activity [65].

Several limitations are worth-noting in the current study. Firstly, the cross-sectional design of this study did not confirm the causality of BMI-for-age and lifestyle behaviors with EF. It could be that adolescents who had poor EF were predisposed to unhealthy behaviors which progressively contributed to obesity. Secondly, large variations in neurocognitive tests across studies might not allow a direct comparison of results. Thus, a more uniform assessment method should be considered in future studies [12]. The EF construct was quite complex; thus, it is suggested that multiple measures for each domain could be used to capture more information, rather than using a single measure. Nevertheless, selection of instruments should be careful and should be based on the age range of a study sample and an ability to separate various domains of EF. Standardized tests may be preferable for enhanced sensitivity with comparison norms provided [66], which has been applied in the current study. Thirdly, the use of self-administered questionnaires may encounter problems of over or underreporting. This problem can be resolved by complementing the study with objective measures. The objective measures may have strengthened the theoretical links that were examined in the present study. For instance, sleep patterns could be captured using polysomnographic recordings, while magnetic resonance imaging could be used to validate neurocognitive test results. On the other hand, the strengths of this study were relatively high participation rates (92%) and separate assessments of EF domains being used. It was clear that sociodemographic factors potentially influenced both obesity and EF. Thus, it was important to control sociodemographic variables when examining the relationships concerning family income, parents’ years of education and household sizes. Furthermore, this study examined the relationship between a wide range of lifestyle behaviors and EF in adolescents, as existing findings only focused on a single lifestyle facet.

## Conclusions

The present findings suggested there was a relationship between obesity and impaired EF among adolescents. Moreover, lifestyle behaviors such as regular meal intakes, higher levels of physical activity and better sleep quality significantly predicted better EF over and above BMI-for-age and sociodemographic influences. Therefore, there is a window of opportunity to improve EF by modifying lifestyle behaviors during adolescence. The current study has provided important information on the relationship between obesity and multiple lifestyle behaviors with EF of adolescents. The present study has also highlighted the need for interventions which target modifiable lifestyle behaviors in order to maximize cognitive benefits, especially during the critical period of brain development of adolescents.

## Supporting information

S1 File Dataset. Data used for the analyses presented in this paper.
(XLSX)
